# *Ex vivo* Platforms to Study the Primary and Recall Immune Responses to Intracellular Mycobacterial Pathogens and Peptide-Based Vaccines

**DOI:** 10.3389/fvets.2022.878347

**Published:** 2022-05-03

**Authors:** William C. Davis, Asmaa H. Mahmoud, Gaber S. Abdellrazeq, Mahmoud M. Elnaggar, John L. Dahl, Victoria Hulubei, Lindsay M. Fry

**Affiliations:** ^1^Department of Veterinary Microbiology and Pathology, Washington State University, Pullman, WA, United States; ^2^Veterinary Quarantine of Alexandria, General Organization for Veterinary Services, Ministry of Agriculture and Land Reclamation, Cairo, Egypt; ^3^Department of Microbiology, Faculty of Veterinary Medicine, Alexandria University, Alexandria, Egypt; ^4^Department of Biology, University of Minnesota Duluth, Duluth, MN, United States; ^5^Animal Disease Research Unit, USDA-ARS, Pullman, WA, United States

**Keywords:** *Mycobacterium tuberculosis*, *Mycobacterium bovis*, *Mycobacterium avium* subspecies *paratuberculosis*, cellular immune response, intracellular pathogens, peptide vaccines

## Abstract

Progress in the study of the immune response to pathogens and candidate vaccines has been impeded by limitations in the methods to study the functional activity of T-cell subsets proliferating in response to antigens processed and presented by antigen presenting cells (APC). As described in this review, during our studies of the bovine immune response to a candidate peptide-based vaccine and candidate *rel* deletion mutants in *Mycobacterium avium paratuberculosis* (*Map*) and *Mycbacterium bovis* (BCG), we developed methods to study the primary and recall CD4 and CD8 T-cell responses using an *ex vivo* platform. An assay was developed to study intracellular killing of bacteria mediated by CD8 T cells using quantitative PCR to distinguish live bacteria from dead bacteria in a mixed population of live and dead bacteria. Through use of these assays, we were able to demonstrate vaccination with live *rel Map* and BCG deletion mutants and a *Map* peptide-based vaccine elicit development of CD8 cytotoxic T cells with the ability to kill intracellular bacteria using the perforin-granzyme B pathway. We also demonstrated tri-directional signaling between CD4 and CD8 T cells and antigen-primed APC is essential for eliciting CD8 cytotoxic T cells. Herein, we describe development of the assays and review progress made through their use in the study of the immune response to mycobacterial pathogens and candidate vaccines. The methods obviate some of the major difficulties encountered in characterizing the cell-mediated immune response to pathogens and development of attenuated and peptide-based vaccines.

## Introduction

*Mycobacterium tuberculosis* (*Mtb*), *Mycobacterium bovis* (*Mbv*), and *Mycobacterium avium* subsp. *paratuberculosis* (*Map*) are members of a lineage of bacteria with a long evolutionary history (1). These bacteria cause tuberculosis in humans and livestock, and paratuberculosis in livestock, respectively. They are representative of multiple lineages of mycobacteria that cause disease in humans and other species. The genetic modifications associated with the adaptation to infect and persist in the vertebrate host were acquired during the evolution of the ancestral *Mycobacterium* that succeeded in establishing a persistent infection in a vertebrate host. When we initiated our studies with *Map*, little was known about the pathogenesis of disease caused by *Map*. Because of the long period of latency before appearance of clinical disease, it was thought there was an age-related difference in susceptibility to infection, and the possibility that exposure didn't always lead to infection. The similarity of paratuberculosis with human and bovine tuberculosis was not clear. Development of flow cytometry and monoclonal antibody reagents for use in cattle made it possible to use cattle as a model species to compare the immune response to *Map* and *Mbv ex vivo*. Initial studies revealed a CD4 and CD8 T-cell response occurs following immunization with either *Map* or *Mbv* (2–4). Exposure to *Map* led to infection of all animals, indicating there was no age-related susceptibility to infection. This observation demonstrated similarity of the immune response to *Map* and other mycobacterial pathogens and indicated comparative studies of the immune response to *Map* and *Mbv* could be conducted to determine whether both organisms utilize the same mechanisms to establish persistent infection.

**Figure 1:**
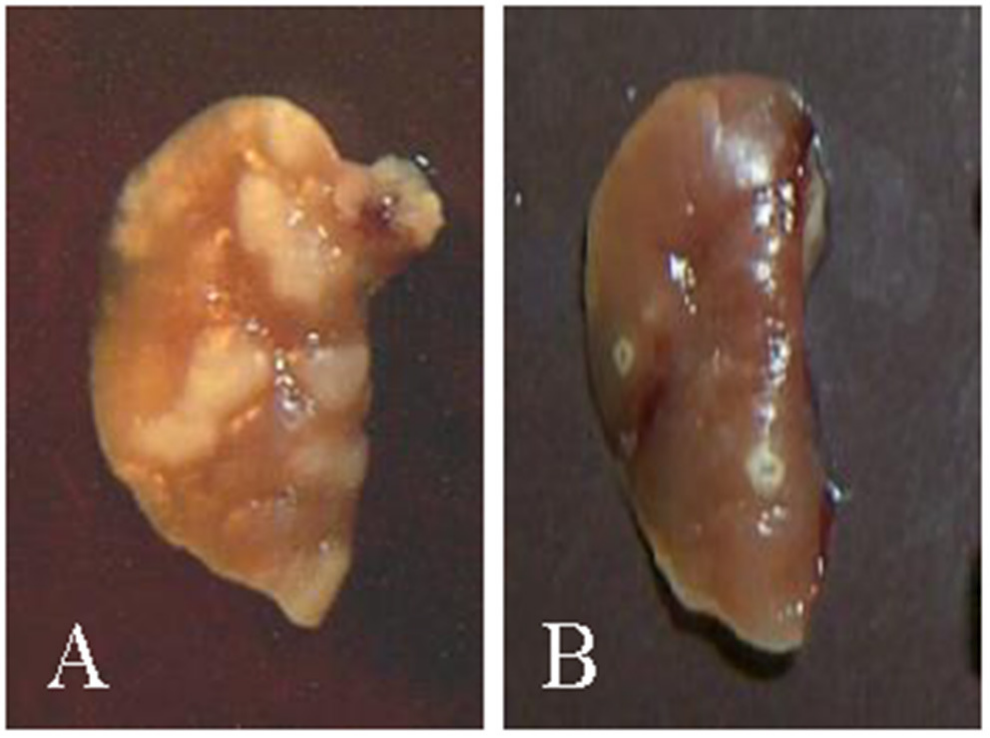
Pictures of lungs obtained from mice at the time of necropsy (6). **(A)** Example of lungs from mice infected with H37rv showing granuloma lesions 3 weeks post infection. **(B)** Example of lungs from mice infected with the H37rv *rel* deletion mutant. Granulomas that developed were cleared by 3 weeks.

Sequencing the genomes of *Mtb, Mbv*, and *Map* afforded opportunities to begin examining the role of specific gene products as virulence factors associated with modulating the immune response, allowing for establishment of a persistent infection. Studies of the stringent response (the ability of bacteria to survive in unfavorable environmental conditions) in *Mtb* in a mouse model provided a lead, suggesting a member of a highly conserved gene system, *rel*, might be involved (5). Comparative analysis of a *rel* deletion mutant in the H37Rv strain of *Mtb* with the wild-type form of H37Rv revealed deletion of the gene resulted in a loss of ability to establish a persistent infection. Pulmonary lesions resolved over time. Histological examination and culture of the lungs showed the bacteria were cleared (Figure 1) (6). At the time, inability of the bacteria to survive was hypothesized to be due to failure of regulatory pathways under control of *rel*, leading to nutrient starvation. The chronology of the infection suggested to us, however, that failure to survive could also be attributable to the development of an immune response that cleared infection of the mutant bacteria. It suggested to us that deletion of *rel* interrupted the mechanisms used by *Mtb* to dysregulate the immune response and establish a persistent infection. It also suggested the finding might be of universal importance if it could be demonstrated that deletion of the gene in another lineage of mycobacteria had the same effect.

To explore this possibility, advantage was taken of improvement of methods to selectively delete genes of interest in *Mtb* (7). The method was optimized for use with slow growing bacteria and used to disrupt *rel* and two other virulence associated genes in *Map, PknG* and *lsr2* (8). *PknG* was selected for comparison to determine if disruption of other virulence-associated genes had the same effect on the capacity to establish a persistent infection (9). Studies were conducted with calves and young goats in different sets of experiments. Analysis of tissues at necropsy showed the presence of *Map* in tissues taken from calves and goats infected with *Map* using culture and PCR. In contrast, analysis of tissues obtained from calves and goats infected with the *Map rel* mutant showed that, as previously observed in mice infected with the *Mtb* H37Rv *rel* deletion mutant bacteria, the mutant bacteria were cleared, and the animals lacked histologic lesions. Comparative studies in calves showed deletion of *PknG* did not prevent establishment of infection (10).

## Development of *Ex vivo* Platforms to Study the Recall and Primary Immune Responses to Candidate Vaccines

**Figure 2:**
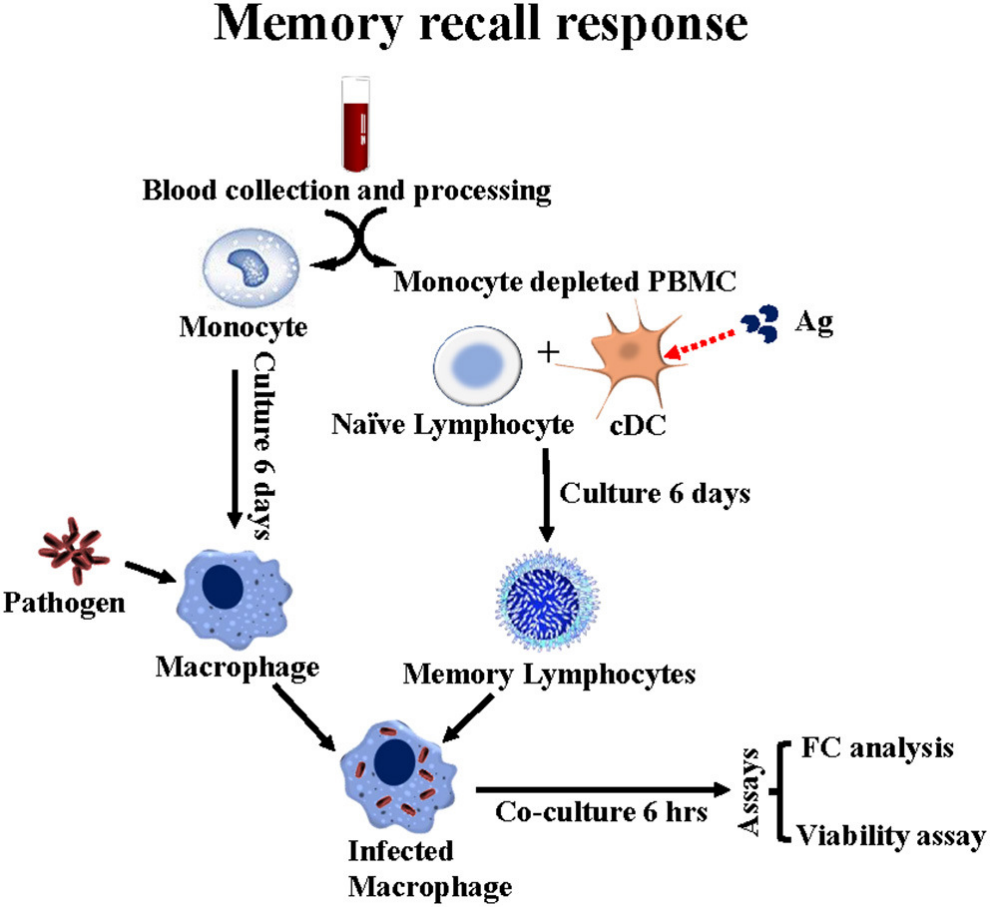
Flow diagram illustrating the *ex vivo* platform developed in cattle to study the recall response to stimulation with APC primed with *Map/rel* or MMP. PBMC from a steer vaccinated with *Map/rel* are separated into CD14^+^ monocytes to develop monocyte derived 14^+^ macrophage target cells and monocyte depleted PBMC for stimulation with CD209^+^ bDC stimulated with *Map/rel* or MMP to elicit a recall response. mdBMC containing bDC, CD4, CD8, CD335 NK cells and γδ T cells are phenotyped by flow cytometry. A viability assay is used to quantitate the relative proportion of bacteria killed by CD8 CTL. See methods for description of assay (13).

The need to necropsy the animals to establish whether deletion of *rel* abrogated the capacity to survive *in vivo* limited opportunities to compare the immune response elicited by wild type *Map* with the immune response elicited by the *rel* mutant. Analysis was only conducted as part of the study with calves. Monoclonal antibodies (mAbs) were used with flow cytometry to characterize the immune response to *Map* and a *Map rel* deletion mutant *Map/rel* (Table 1). A tissue culture platform was developed to examine the potential role of the cell-mediated immune response in preventing establishment of a persistent infection (Figure 2) (11). Development of a mAb to CD209, a molecule specifically expressed by dendritic cells (DC) in cattle, provided an opportunity to look at the recall response to *Map* antigens (Ag) processed and presented by three types of antigen presenting cells (APC): DC present in blood (bDC), monocyte-derived DC (MoDC), and monocyte-derived macrophages (MoMΦ) (11). A consistent observation obtained with PBMC from a steer vaccinated with *Map/rel* was that direct stimulation of PBMC and PBMC depleted of monocytes (mdPBMC) with *Map/rel*, MoDC or MoMΦ, primed with *Map/rel*, elicited the same proliferative response in CD4 and CD8 CD45R0 positive memory T cells (11). Further studies were conducted to determine the target of the immune response. A 35 kDa major membrane protein (MMP) was selected as the first candidate to examine based on previous studies that demonstrated it plays a role in invasion of bovine epithelial cells (12). MMP elicited an identical recall response. A final set of experiments were conducted to determine if the recall response was MHC-restricted. Pre-incubation of MoDC and MoMΦ with mAb specific for MHC class I and class II molecules blocked the proliferative recall T-cell response, demonstrating that the response was MHC-restricted (11).

**Table 1:** mAbs used to study the immune response to mycobacterial pathogens.

**mAb**	**Isotype**	**Specificity**
H58A	IgG2a	MHC I
PT85A	IgG2a	MHC I
TH14B	IgG2a	MHC II HLA-DR orthologue
TH81A	IgG2a	MHC II HLA-DQ orthologue
IL-A11A	IgG2a	CD4
CACT138A	IgG1	CD4
7C2B	IgG2a	CD8
IL-A116A	IgG3	CD8
CACT116A	IgG1	CD25
LCTB2A	IgG3	CD25
GB21A	Igg2b	γδ TCR δ chain specific
EC1.1	IgG1	CD335 NK cells
CAM36A	IgG1	CD14
DH59B	IgG1	CD172a
209MD26A	IgG2a	CD209

*The mAbs were obtained from the WSU Monoclonal Antibody Center*.

**Figure 3:**
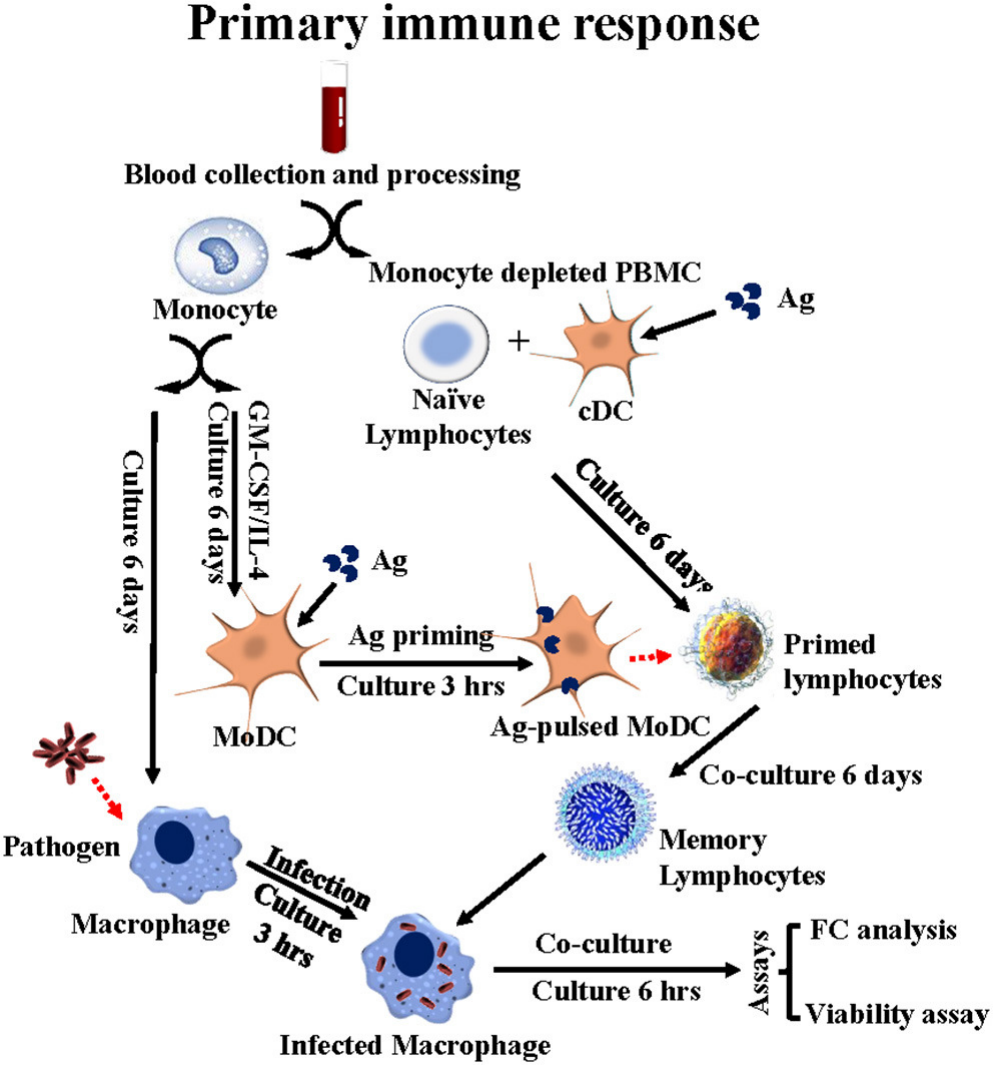
Flow diagram illustrating the *ex vivo* platform developed in cattle to study the primary immune response to stimulation with APC primed with Ag. PBMC from a unvaccinated steer are separated into monocytes and monocyte-depleted PBMC (mdPBMC). The monocytes are stimulated with GM-CSF and IL-4 to generate CD209^+^ MoDC. The mdPBMC, containing CD209^+^ bDC, are stimulated with Ag. At 6 days the MoDC are primed with Ag and used to restimulate the primed mdPBMC. A second set of monocytes are isolated and cultured to generate macrophage target cells. At 6 days post stimulation with Ag primed MoDC, the mdPBMC are mixed with the infected target cells. At 6 or 24 h, FC is used to phenotype the CD45R0^+^ memory lymphocytes. The bacteria viability assay is used to determine the extent of killing of intracellular bacteria mediated by CD8 CTL.

Results obtained with the *ex vivo* culture system suggested it might be possible to look at the primary response as well as the recall immune response *ex vivo*, and thereby characterize the functional activity of CD4 and CD8 T cells, γδ T cells and NK cells stimulated with Ag-primed APC. MMP was used to conduct these studies. Two rounds of stimulation were necessary to obtain enough cells for analysis. bDC in mdPBMC were used in the first round of stimulation and MoDC in the second round of stimulation (Figure 3). CD4 and CD8 T cells were the main cell types present in primary cultures of mdPBMC, consistent with results obtained in study of the recall response. Little or no proliferative response of γδ T cells or NK cells was evident in cultures of mdPBMC stimulated with *Map/rel-*primed APC (13).

## Development of an Assay to Characterize the Functional Activity of Antigen Specific CD4 and CD8 T Cells

Studies conducted with bacille Calmette-Guérin (BCG) in humans by Worku and Hoft (14) suggested an *ex vivo* assay could be developed to study the functional activity of CD4 and CD8 T cells proliferating in response to stimulation with *Map/rel-*primed APC. They used MoMΦ infected with BCG as targets, and tritiated uridine and the colony forming unit (CFU) assays to assess the effect of co-culture of PBMC from BCG-vaccinated humans on the intracellular growth of BCG. They used flow cytometry to monitor a change in size of lymphocytes using side vs. forward light scatter (SSC vs. FSC), associated with the proliferative response to stimulation with BCG and mycobacterial antigens (Ag). Cells were concurrently assessed using flow cytometry and anti-CD4, -CD8, -γδ T cell antibodies to determine the identity and frequency of cell subsets proliferating in response to stimulation with MoMΦ primed with BCG or mycobacterial Ags. Their studies demonstrated PBMC from latently infected and vaccinated humans stimulated with BCG and Ag-primed MoMΦ inhibited growth of intracellular BCG. PBMC stimulated with irrelevant Ags enhanced intracellular survival. Similar studies were conducted more recently by Pooley et al. (15) using the methods developed by Worku and Hoft to study the immune response to *Map* in sheep. In their studies, PBMC were collected and separated into mdPBMC and monocyte adherent cells. The mdPBMC were rested overnight. The monocyte adherent cells target cells were incubated with *Map* overnight and washed the next day prior to mixing with the mdPBMC. The mdPBMC were washed away. A rapid culture qPCR was used to assess killing (16). The assay showed there was a reduction in viable bacteria obtained from monocyte infected target cells mixed with mdPBMC from infected and vaccinated sheep.

**Figure 4:**
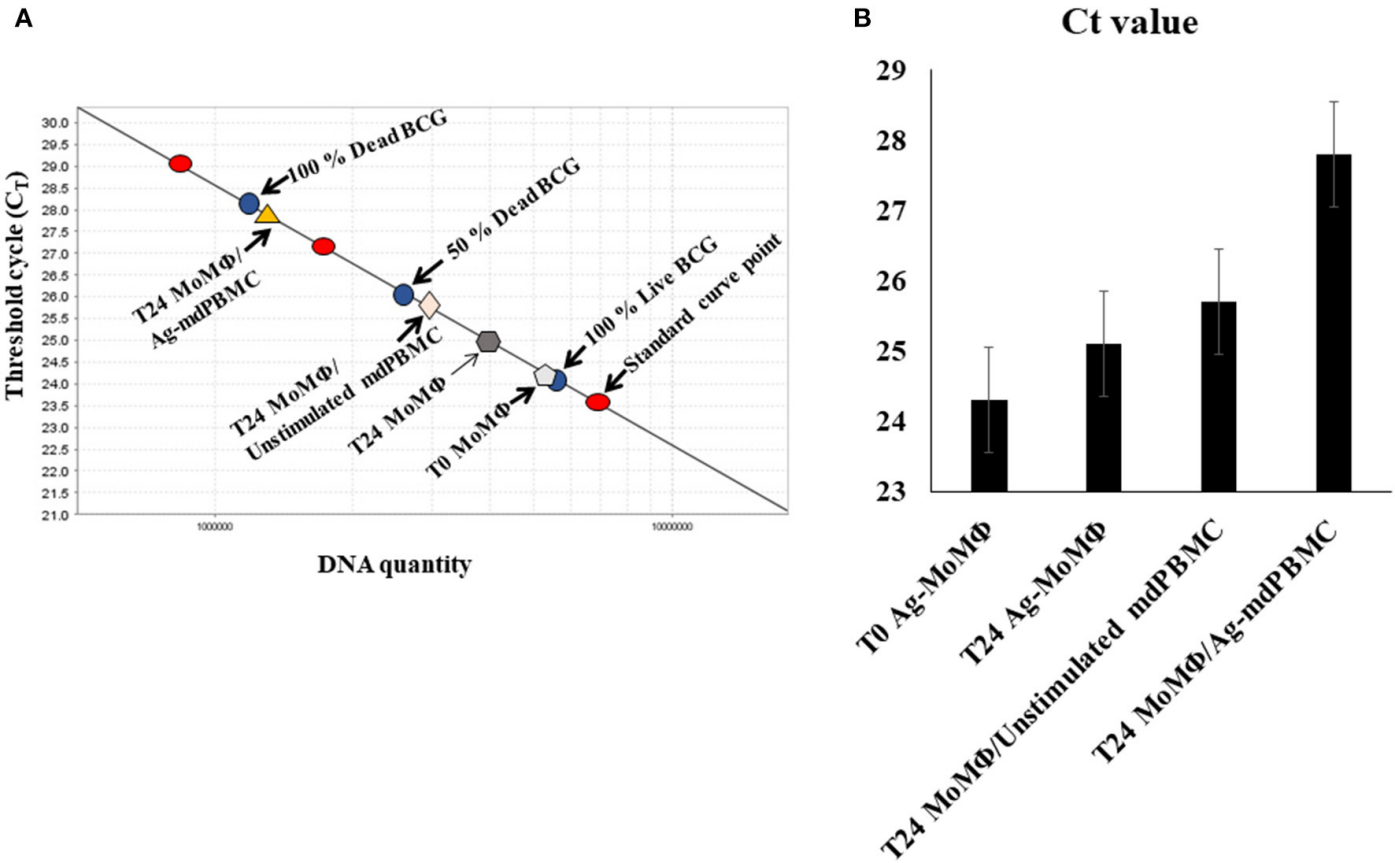
Illustration of the quantitative PCR method using propidium monoazide (PMA) and a single copy gene from *Map*, F57, or *lpqT* (Rv1016c) from Mbv BCG to distinguish live from dead bacteria. **(A)** A standard curve is generated from a single copy gene. A known concentration of DNA from live bacteria (100%), a mixture of 50% live bacteria and 50% dead bacteria, and 100% dead bacteria are used to show the range of sensitivity for quantifying the proportion of intracellular bacteria killed by CD8 CTL. Controls include the percent of live bacteria present in MoM*Φ* 3 h after uptake by MoM*Φ*, 24 h after uptake, and 24 h after incubation of MoM*Φ* with unstimulated mPBMC. Covalent binding of propidium monoazide to DNA is incomplete but sufficient for allowing comparison of the differences in the percent of DNA from live and dead bacteria from infected MoM*Φ* target cells in relation to the DNA detected in the 100% live, 50% live and 50% dead, 100% dead control (13, 23). **(B)** Histogram illustrating summary results of *C*_t_ values obtained from standard curve. T0 in **(B)** refers to infected target MoM*Φ* pre-incubated with bacteria 3 h before use (13).

The assays used to examine the effect of Ag-primed PBMC on survival of intracellular bacteria limited the ability of Worku and Hoft to determine the mechanisms used by Ag- specific T cells to inhibit the intracellular growth of bacteria. The method used by Pooley et al. also limited the ability to examine the mechanisms of killing. Concurrent studies by Kralik et al. (17) were focused on developing a more direct way to circumvent the limitations of the CFU assay. Through use of a membrane impermeable viability dye, propidium monoazide, in combination with a quantitative PCR, using a single copy *Map* specific gene (F57), they were able to demonstrate live bacteria could be distinguished from dead bacteria in a mixed population of dead and live bacteria. We adapted the method for use in distinguishing live from dead bacteria obtained from MoMΦ target cells infected with *Map* (Figure 4) (13).

## Functional Activity of CD4 and CD8 T cells Proliferating in Response to Stimulation With MMP-Primed APC

Modification of the method developed by Kralik et al. demonstrated that bacteria could be isolated from MoM*Φ* target cells and processed to distinguish live from dead bacteria (17). This provided an opportunity to examine the functional activity of CD4 and CD8 T cells that develop in response to stimulation with MMP-primed APC. Use of the method revealed killing of intracellular bacteria could be detected within 6 h of mixing MMP-stimulated mdPBMC with *Map*-infected MoM*Φ* target cells. Reciprocal depletion studies demonstrated killing was mediated by CD8 T cells. Any killing activity associated with CD4 T cells was below the level of detection. Consistent with previous observations, little or no proliferative or CTL activity was detected in γδ T cells present in cultures of mdPBMC. The frequency of NK cells was invariably low, limiting opportunities to determine if they had any CTL activity.

The ability to examine the functional activity of CD8 T cells *ex vivo* also provided an opportunity to examine the mechanism used by CD8 T cells to kill intracellular bacteria. Concurrent studies by Dotiwala et al. (18) and Walch et al. (19) had reported intracellular killing is mediated through the perforin, Granzyme B, granulysin pathway. Flow cytometry was used to examine the phenotype of CD4 and CD8 stimulated with MMP-primed APC before and after mixing with infected target cells. Intracellular labeling demonstrated the presence of perforin in cytotoxic granules present in both CD4 and CD8 T cells in unstimulated lymphocytes. Use of a mAb that distinguished memory T cells from naïve T cells showed detectable perforin was present in equal proportion in naïve and memory T cells prior to mixing with infected target cells. There was no apparent increase in perforin in naïve and memory CD4 T cells following mixing with infected target cells. In contrast, there was a clear increase in perforin in naïve and memory CD8 T cells following mixing with infected target cells with almost all of the memory CD8 T cells containing perforin. Labeling to detect Granzyme B revealed approximately half the CD8 memory T cells expressed Granzyme B. A mAb specific for granulysin was not available at the time of the studies (13).

## Both BCG and a BCG/*rel* Deletion Mutant Elicit Development of CD8 CTL

The *ex vivo* platform also provided an opportunity to examine the immune response to BCG and determine whether deletion of *rel* might improve the efficacy of BCG as a vaccine. The use of BCG as a vaccine in humans has shown it is not fully effective (20). Extensive studies in cattle have shown it is not effective as a vaccine in its present form (21, 22). Efforts to increase its efficacy through further genetic modification has not been successful. Our studies suggested the reason for limited efficacy might be associated with BCG being able to establish a persistent infection (20). A *rel* deletion mutant was developed in BCG and used to compare the immune response of BCG/*rel* to BCG (23). No difference was detectable in the proliferative response of CD4 and CD8 T cells primed with BCG or BCG/*rel*. Intracellular killing was similar and dramatic. Infected target cells lost adherence to the surface of the culture plates within minutes following addition of CD8 CTL. Further analysis showed the perforin, Granzyme B, and granulysin pathway was used in killing (23). As observed with the studies of *Map*, development of CD8 CTL was MHC-restricted (23). The finding that BCG also elicits development of CD8 CTL was expected since vaccination with BCG does elicit an immune response that has some protective effect against *Mbv*. Further studies are needed to determine how the immune response to the *rel* deletion mutant differs from the immune response to BCG.

## TRI-Directional Signaling is Essential for Eliciting CD8 Cytotoxic T cells

**Figure 5:**
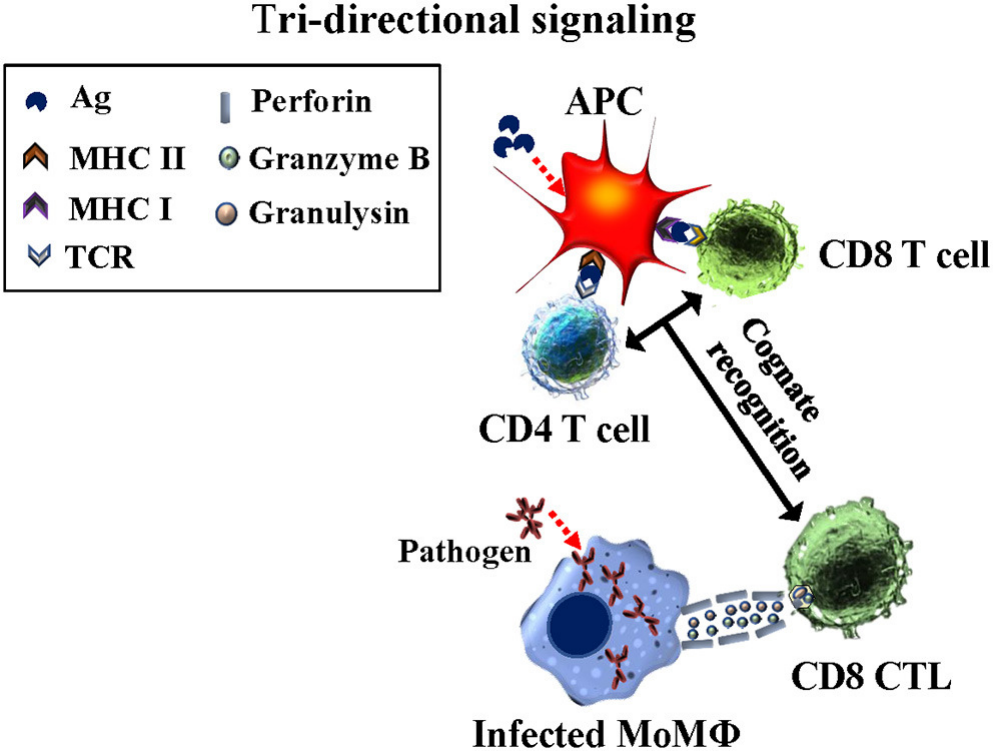
Diagram illustrating the concurrent tri-directional signaling required for development of CD8 CTL during the primary immune response and eliciting a CD8 CTL recall response. Antigen must be taken up and processed by APC for presentation in context of MHC I and MHC II molecules. CD4 and CD8 T cells must recognize their respective epitopes concurrently interfaced with the APC. Reciprocal signaling occurs from the APC to CD4 and CD8 T cells. Essentiall signaling also occurs between CD4 and CD8 T cells while in contact with the APC (25).

Development of the *ex vivo* platform to study primary and recall T-cell responses provided an opportunity to investigate another issue impeding vaccine development progress, especially peptide-based vaccines. Extensive studies had shown CD4 T cells are essential for development of long-lived CD8 memory T cells. Detailing the signaling between CD4 T and CD8 T cells essential for eliciting development of CD8 CTL has been elusive [reviewed in (24)]. Model systems used to determine how and when T cell help is delivered to elicit development of CD8 CTL have not yielded definitive information. Studies with the *Map/rel* and *BCG/rel* mutants provided data showing CD4 T cells must be present in cultures with Ag-primed APC for development of CD8 CTL. The studies also demonstrated stimulation with Ag-primed APC is MHC-restricted. The complexity of the immune response to live bacteria, however, made it difficult to determine antigenic features required to elicit a CTL response and also, the necessary timing of signaling interactions between CD4 and CD8 T cells and Ag-primed APC for development of CTL. As in our studies with *Map/rel* and *BCG/rel* mutants, studies with MMP also demonstrated CD4 T cells must be present for eliciting CD8 CTL (Figure 5). The use of mAbs specific for MHC I and II molecules provided a way to look specifically at the timing of the interaction between CD4 and CD8 T cells with Ag-primed APC. An experiment was designed to look at the primary immune response to the MMP Ag *ex vivo* to reduce the complexity of the immune response to a single Ag. The experiment was conducted in the presence and absence of mAbs specific for MHC I and MHC II. As previously observed, pre-exposure of Ag-primed APC to both anti-MHC I and II mAbs at the same time blocked development of CTL. Pre-exposure to MHC I or MHC II mAbs alone also blocked development of CTL. Blocking of MHC II alone demonstrated CD4 T cells could not be primed by the Ag-primed APC to deliver CD4 help, because the CD4 T cells could not make contact with the APC. Blocking of MHC I showed CD4 T cells could be primed by interacting with the APC but the CD8 T cells could not make contact with the APC and receive help from the primed CD4 T cells associated with the APC. Further studies need to be conducted, but the results indicate tri-directional signaling must occur for development of CTL (25). The results also show that a peptide antigen must contain epitopes recognized by MHC I and II to elicit CD8 CTL. This has been difficult to demonstrate with other experimental approaches [reviewed in (24)].

## A Universal Platform for Study of the Immune Response to Pathogens and Candidate Vaccines

Cattle have proven useful as a model species for development of an *ex vivo* platform to investigate how pathogens have evolved to modulate the immune response and cause disease, information that is crucial to vaccine development. Importantly for comparative studies, they are phylogenetically more closely related to humans, with higher genetic identity of genes involved in regulation of the immune response than mice. This expands opportunities for more direct comparisons of the immune response to pathogens affecting humans, cattle, and other species. Because of their size, longevity, and large blood supply, multiple studies can be conducted with a single animal. This reduces variables associated with differences in the MHC and differences in other genes that regulate development of an immune response. Conversely, multiple animals with different MHC class I and class II haplotypes can be utilized when differences in MHC type must be evaluated. Monoclonal antibodies have been identified that recognize highly conserved epitopes expressed on orthologues of MHC class I and class II, HLA-DR and HLA-DQ, in multiple species, facilitating cross-species comparisons with the same reagents (26). Monoclonal antibodies have also been developed or identified that recognize molecules expressed on the major subsets of lymphocytes, NK cells, monocytes/macrophages, DC, and granulocytes. The mAbs make it possible to include ruminants, especially cattle and water buffalo as outbed model species for comparative studies of pathogens affecting livestock and human health (27). The mAbs are available through the non-profit Washington State Monoclonal Antibody Center Washington State University – Monoclonal Antibody Service Center (Washington State University – Monoclonal Antibody Service Center (wsu.edu) and national and international distributors.

We have focused on use of the platform for the study of mycobacterial pathogens because of their importance to animal and human health at the national and international level. *Mtb, Mbv*, and *Map* are representative of multiple lineages of mycobacteria that cause disease in humans and other species. Progress in developing vaccines has been slow because of a lack of understanding of how the pathogens establish a persistent infection. Infection leads to development of an immune response that controls, but is unable to clear the pathogen, allowing for development of latent (persistent) infection. The complete details as to how mycobacteria persist in the presence of an immune response remain elusive. However, studies completed thus far provide a lead that may prove to be of universal importance for mycobacteria sp. and other pathogenic bacteria (23).

RelA (RelA/SpoT homologue) is a member of a highly conserved group of proteins with sequence similarity to the RelA and SpoT enzymes of *Escherichia coli* that comprise a superfamily of enzymes that synthesize and/or hydrolyze the alarmone signaling nucleotide ppGpp, (p)ppGpp, activator of the stringent response [reviewed in (28)]. The homologue in gram positive bacteria is referred to as Rel [reviewed in (5, 28)] Point mutations in *rel* in *Mtb* have shown the gene encoding a synthetase is essential for mediating the stringent response [reviewed in (29)]. Studies by Dutta et al. (30) have shown inhibition of the stringent response blocks entry of *Mtb* into the quiescent state and reduces persistence. The main interpretation of the effect of deleting *rel* on survival of pathogenic bacteria, including mycobacteria, has been that nutrient starvation in the vertebrate host accounts for the loss of ability to survive in the vertebrate host (5). Our studies show loss of ability to survive in the vertebrate host includes development of CD8 T cells with the ability to kill intracellular bacteria. These results may be of universal importance; suggesting *rel* is the Achilles' heel for multiple lineages of pathogenic bacteria. Further studies in other lineages of bacteria are clearly needed. A search of the literature yielded information on only one other group of investigators studying the effect of deleting *rel* in *Francisella novicida* (31). Similar to studies by Dahl et al. (6) demonstrated survival of the *rel* mutant in a mouse model was reduced. A challenge study conducted at the same time, with the mice immunized with the *rel* mutant, showed an immune response developed that reduced survival of wild type *F. novicida* used for challenge (31).

In summary, methods developed to study the immune response to mycobacterial pathogens overcome some of the challenges of studying the immune response to pathogens and the development of vaccines. Reagents are now available through the WSU Monoclonal Antibody Center and distributers that make it possible to use cattle as a model species for research. It is hoped that sufficient detail has been provided to facilitate use of the methods by other investigators.

## Author Contributions

WD developed the initial draft of the review. AM, GA, ME, VH, and LF participated in writing the final manuscript. All authors have read and agreed to the published version.

## Funding

The studies reviewed in this manuscript were supported in part by the National Institute of Food and Agriculture-Agriculture and Food Research Initiative Competitive Grant 2018-67015-28744 (WD). Mention of trade names, proprietary products, or specified equipment do not constitute a guarantee or warranty by the USDA and does not imply approval to the exclusion of other products that may be suitable. USDA is an Equal Opportunity Employer. This review was also supported in part by the WSUMAC: http://vmp.vetmed.wsu.edu/resources/monoclonal-antibody-center.

## Conflict of Interest

The authors declare that the research was conducted in the absence of any commercial or financial relationships that could be construed as a potential conflict of interest.

## Publisher's Note

All claims expressed in this article are solely those of the authors and do not necessarily represent those of their affiliated organizations, or those of the publisher, the editors and the reviewers. Any product that may be evaluated in this article, or claim that may be made by its manufacturer, is not guaranteed or endorsed by the publisher.
